# Distribution, quantification, and characterization of substance P enteric neurons in the submucosal and myenteric plexuses of the porcine colon

**DOI:** 10.1007/s00441-023-03842-x

**Published:** 2023-11-20

**Authors:** Maurizio Mazzoni, Luis Cabanillas, Anna Costanzini, Filippo Caremoli, Mulugeta Million, Muriel Larauche, Paolo Clavenzani, Roberto De Giorgio, Catia Sternini

**Affiliations:** 1 Department of Veterinary Medical Sciences, University of Bologna, Ozzano Emilia, 40064 Bologna, Italy; 2 Division of Digestive Diseases, Department of Medicine, David Geffen School of Medicine, UCLA, Los Angeles, CA 90095 USA; 3 Department of Neurobiology, David Geffen School of Medicine, UCLA, Los Angeles, CA 90095 USA; 4 Department of Translational Medicine, University of Ferrara, Ferrara, Italy; 5 Current address: San Raffaele Hospital, Milan, Italy; 6 Department of Integrative Biology & Physiology, UCLA, Los Angeles, CA 90095 USA

**Keywords:** Cholinergic and nitrergic transmission, Excitatory motor neurons, Inhibitory motor neurons, Secretomotor neurons, Interneurons

## Abstract

The pig is an important translational model for studying intestinal physiology and disorders for its many homologies with humans, including the organization of the enteric nervous system (ENS), the major regulator of gastrointestinal functions. This study focused on the quantification and neurochemical characterization of substance P (SP) neurons in the pig ascending (AC) and descending colon (DC) in wholemount preparations of the inner submucosal plexus (ISP), outer submucosal plexus (OSP), and myenteric plexus (MP). We used antibodies for the pan-neuronal marker HuCD, and choline acetyltransferase (ChAT) and neuronal nitric oxide synthase (nNOS), markers for excitatory and inhibitory transmitters, for multiple labeling immunofluorescence and high-resolution confocal microscopy. The highest density of SP immunoreactive (IR) neurons was in the ISP (222/mm^2^ in the AC, 166/mm^2^ in the DC), where they make up about a third of HuCD-IR neurons, compared to the OSP and MP (19–22% and 13–17%, respectively, *P* < 0.001–0.0001). HuCD/SP/ChAT-IR neurons (up to 23%) were overall more abundant than HuCD/SP/nNOS-IR neurons (< 10%). Most SP-IR neurons contained ChAT-IR (62–85%), whereas 18–38% contained nNOS-IR with the highest peak in the OSP. A subpopulation of SP-IR neurons contains both ChAT- and nNOS-IR with the highest peak in the OSP and ISP of DC (33–36%) and the lowest in the ISP of AC (< 10%, *P* < 0.001). SP-IR varicose fibers were abundant in the ganglia. This study shows that SP-IR neurons are functionally distinct with variable proportions in different plexuses in the AC and DC reflecting diverse functions of specific colonic regions.

**Supplementary Information:**

The online version contains supplementary material available at 10.1007/s00441-023-03842-x.

## Introduction

The enteric nervous system (ENS), also known as the “brain in the gut,” has a primary role in maintaining body homeostasis through constant regulation of different gastrointestinal (GI) functions including motility, secretion, blood flow, and ion transport (Furness 2012; Furness et al. 2014). The ENS is composed of millions of neurons embedded in the wall of the GI tract and highly organized in two main ganglionated plexuses, the myenteric plexus (MP), situated between the longitudinal and circular muscle layers, and the submucosal plexus, located in the submucosa, whose structural organization depends on the animal species. Indeed, the ENS in large animals, such as humans and pigs, has a multilayered submucosal plexus with an inner submucosal plexus (ISP) near the mucosa and an outer submucosal plexus (OSP) near the circular muscle (Timmermans et al. 1992, 1997; Brown and Timmermans 2004; Furness 2012; Petto et al. 2015; Mazzoni et al. 2021). The pig is a valuable model for studying intestinal functions and disorders for its homologies with humans, such as microbiome composition, size, nutrition (being both omnivores and colon fermenters), and the ENS organization (Miller and Ullrey 1987; Brown and Timmermans 2004; Pang et al. 2007; Bassols et al. 2014). Enteric neurons exhibit a high degree of differentiation in terms of morphology, functions, electrophysiological properties, and neurochemical characterization, a feature preserved in different species (Timmermans et al. 1990, 1992; Furness 2000).

In our previous study (Mazzoni et al. 2021), we provided a thorough evaluation of enteric neuron density in the different plexuses in the ascending and descending regions of the pig colon as well as the relative abundance of enteric neurons containing immunoreactivities for choline acetyltransferase (ChAT) and neuronal nitric oxide synthase (nNOS), markers for excitatory and inhibitory enteric neurons, respectively. In this study, we focused on the quantification of enteric neurons containing substance P (SP) immunoreactivity (IR) and their neurochemical characterization. SP is an undecapeptide belonging to the family of tachykinin neuropeptides, which share high sequence homology, i.e., the region Phe-X-Gly-Leu-Met-NH2 where X is an aromatic or hydrophobic residue required for biological activity (Sternini et al. 1989; Shimizu et al. 2008). Though we elected to use the term SP immunoreactity to describe the staining obtained with our SP antibody for simplicity, it should be kept in mind that SP immunostaining could be attributable to other tachykinins. SP immunoreactivity has been identified in enteric neuronal perikarya within intramural plexuses, as well as in nerve processes, which innervate intestinal muscles and submucosal and mucosal layers of several parts of the mammalian GI tract in different species, including the pig (Timmermans et al. 1989; Schmidt et al. 1991; Hens et al. 2000; Brookes 2001; Brehmer et al. 2004; Mitsui 2011; Gonkowski 2013; Petto et al. 2015; Filmayer et al., 2020). SP is a neurotransmitter/neuromodulator, which modulates motility patterns, vascular tone, and some immunological aspects (Holzer and Holzer-Petsche 1997a, b). Specifically, SP cooperates with acetylcholine in evoking a strong excitatory effect on the smooth muscle in any segment of the GI tract (Holzer and Holzer-Petsche 1997a, b; Koon and Pothoulakis 2006). In addition to these canonical effects, evidence indicates that SP functions as a pro-inflammatory mediator released from sensory nerves, myenteric neurons, and inflammatory cells (Koon and Pothoulakis 2006). In order to establish the neurochemical profile of SP-containing neurons in the ENS of the pig colon, we used wholemount preparations to enable quantitative analysis with multiple labeling immunofluorescence, high-resolution confocal microscopy and Imaris software for quantification.

## Material and methods

### Animals

A total of 23 animals, 7 months old (12 h fasted), 20 castrated (on post-natal day 7) male and 3 intact female Yucatan minipigs, with a body weight of 25–30 kg were used. Animal care and procedures described in this study were carried out in strict accordance with the National Institutes of Health recommendations for the humane use of animals. The experimental procedures were approved by the University of California, Los Angeles (UCLA), Chancellor’s Animal Research Committee (ARC) (protocol 2018–074﻿01). Pigs were obtained from S&S Farms (Farms, Ramona, CA) and group housed in pens in an environmentally controlled room (lights on/off 6 AM/6 PM, 61–81 °F). Pigs had free access to food (5p94 Prolab mini pig diet, PMI Nutrition, St. Louis, MO) and filtered tap water.

All animals were pre-medicated with intramuscular midazolam (1 mg/kg; cat # 067595, Covetrus, Dublin, OH), ketamine (15 mg/kg; # 068317, Covetrus, Dublin, OH), and meloxicam (0.3 mg/kg; # 049755, Covetrus, Dublin, OH) and euthanized with an intravenous injection of pentobarbital (100 mg/kg, cat # 009444, Covetrus). In some animals, where colonic motility was recorded by luminal manometry probes, tissues were collected 5 h post induction of anesthesia, whereas in other animals, specimens were collected immediately after induction of anesthesia. A pilot analysis did not reveal any differences in the total density of enteric neurons and the localization of the investigated neuronal markers between specimens collected immediately or 5 h following anesthesia (Mazzoni et al. 2021). From each animals, specimens were collected from the ascending colon (AC) in correspondence to the central flexure, and the descending colon (DC), about 20–30 cm from the anus.

### Tissue preparation

Colonic specimens were immersed in 0.01 M phosphate buffer saline (PBS, pH 7.0) containing the L-type calcium channel blocker, nicardipine (20 mM), for 15–40 min. The tissues were opened along the mesenteric border, washed with PBS, and pinned tightly on balsa wood. Specimens were then fixed in Zamboni’s fixative (2% paraformaldehyde containing 0.2% picric acid in PBS) at 4 °C overnight, removed from the balsa wood, flashed 3 times (10 min each) in dimethyl-sulfoxide (DMSO, Sigma-Aldrich), followed by washing in PBS and stored at 4 °C in PBS containing sodium azide (0.1%). Wholemount preparations of the MP were obtained by separating the longitudinal muscle layer with attached the MP from the submucosa and mucosa using a dissecting microscope. The mucosa was removed from the submucosa and the submucosa further separated into the inner (ISP) and outer (OSP) parts of the submucoal plexus.

### Immunohistochemistry

Wholemount preparations for each plexus (ISP, OSP, and MP) were obtained from the AC and DC specimens collected from each animal and processed for immunohistochemistry. In order to establish the total number of neurons in each plexus and the distribution of subclasses of neurons, HuCD, ChAT, nNOS, and SP primary antibodies were used (Table 1). Initial single labeling immunofluorescence experiments determined each individual antibody best dilution and incubation time. Wholemount preparations were incubated in 10% normal goat serum (NGS, Sigma-Aldrich) in PBS containing 3% Triton-X100 and 1% BSA for 1 h at room temperature to reduce nonspecific binding of the secondary antibodies and to permeabilize the tissue to the antisera. Primary antibodies were diluted in PBS containing 1% Triton-X100 and 3% NGS; secondary antibodies were diluted in PBS containing 3% NGS. Tissues were then incubated at 4 °C in a humid chamber for 48 h with the primary antibodies, washed in PBS (4 × 10 min), and incubated for 3 h at room temperature in a humid chamber in a solution containing the secondary antibody (e.g., goat anti-mouse Alexa Fluor^®^ 594 or 405) (Table 1). For double and triple labeling experiments, we used the sequential staining method (Ho et al. 2003; Mazzoni et al 2021), which we have shown to result in the best staining/background ratio. Also, this procedure allows the use of two mouse monoclonal antibodies in the same preparation. For double labeling, tissues were incubated with the first primary antibody (e.g., rabbit anti-ChAT), followed by goat anti-rabbit Alexa Fluor^®^ 488, then incubated with the second primary antibody (e.g., mouse anti-HuCD) followed by goat anti-mouse Alexa Fluor^®^ 594. Monoclonal antibodies to nNOS and HuCD and polyclonal rabbit anti-ChAT, rabbit anti-nNOS, and rat anti-SP antisera have been previously validated (Hens et al. 2000; Murphy et al. 2007; Petto et al. 2015; Mazzoni et al. 2021).

**Table 1:** List of primary and secondary antibodies and respective dilutions

Marker	Code	Case product	Dilutions
Mo anti-nNOS	SC-5302	Santa Cruz	1:100
Rb anti-nNOS	ab15203	Abcam	1:100
Rb anti-ChAT	P3YEB	(Prof. Schemann)	1:800
Mo anti-HuCD	A-21271	Thermofisher Scientific	1:100
Rat anti-SP	10-S15A	Fitzgerald	1:1000
Secondary antibody			
Goat anti-mouse Alexa Fluor^®^ 594	A11032	Thermofisher Scientific	1:800
Goat anti-rabbit Alexa Fluor^®^ 488	A11008	Thermofisher Scientific	1:2000
Goat anti-mouse Alexa Fluor^®^ 488	A11029	Thermofisher Scientific	1:1000
Goat anti mouse Alexa Fluor ^®^405	A31553	Thermofisher Scientific	1:1000
Goat anti-mouse Alexa Fluor ^®^ 633	A21052	Thermofisher Scientific	1:1000
Goat anti-rat Alexa Fluor ^®^ 488	A11006	Thermofisher Scientific	1:1000
Goat anti-rat Alexa Fluor ^®^ 594	A11007	Thermofisher Scientific	1:1000
Goat anti-rat Alexa Fluor ^®^ 633	A21094	Thermofisher Scientific	1:1000
Goat anti-rat Alexa Fluor ^®^ Plus 594	A48264	Thermofisher Scientific	1:1000

### Quantitative analysis of ISP, OSP, and MP neurons

Wholemount preparations were examined using Zeiss LSM 880 Fast-Airyscan confocal microscope for image analysis and the Imaris software (Imaris for Neuroscientists) for quantification. In order to comprehensively account for all cells containing specific staining taking into consideration different fluorescent gradients, we initially established a threshold for immunofluorescence detection based on intensity and cell size with Imaris. In addition, immunostaining for each antibody combination was evaluated by two observers, and if there were inconsistencies or discordances between the observers, additional evaluations were conducted comparing immunoreactive neurons in multiple images to eliminate possible false positive and only neurons for which both observers agreed were included. To determine the total number of neurons, the HuCD immunoreactive (-IR) neurons were counted. The number and percentage of SP/IR neurons were expressed as the number of neurons per mm^2^ and the percentage of the total number of HuCD-IR neurons. In addition, we evaluated the percentage of HuCD/SP/IR neurons that co-expressed ChAT or nNOS (i.e., the following combinations: SP/ChAT/HuCD, ChAT/SP/HuCD, SP/nNOS/HuCD, and nNOS/SP/HuCD) and the percentage of SP-IR neurons that expressed both ChAT- and nNOS-IR (SP/ChAT/nNOS, ChAT/nNOS/SP, and nNOS/ChAT/SP). Data were expressed as means ± standard error of the mean (SEM). One-way and two-way ANOVA followed by Bonferroni post-test for multiple comparisons were used for statistical analysis (*P* < 0.05 for significance). GraphPad Prism software version 8.3.0 for Windows (GraphPad Software, San Diego, CA) was employed for these analyses and graphing.

## Results

### Density of SP-IR neurons in the ISP, OSP, and MP

**Fig. 1:**
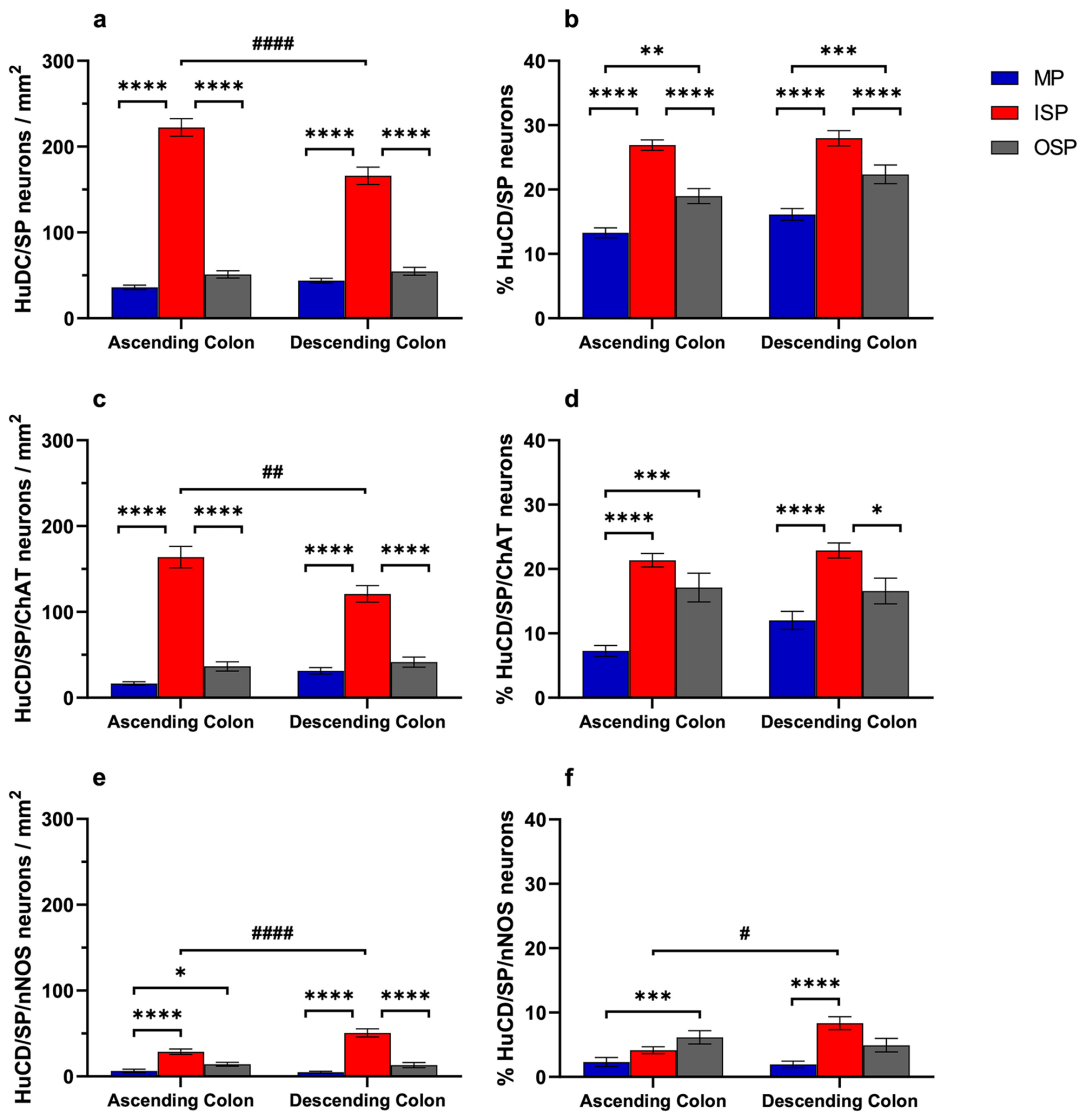
Graphs showing the density of neurons containing HuCD/SP-immunoreactivity (IR) (**a**, **b**), HuCD/ChAT/SP-IR (**c**, **d**), or HuCD/SP/nNOS-IR (**e**, **f**) in the inner submucosal plexus (ISP), outer submucosal plexus (OSP), and myenteric plexus (MP) of the ascending and descending colon. Neuronal density is expressed as numbers of neurons/mm^2^ (**a**, **c**, **d)** and % of neurons visualized with HuCD-IR (**b**, **d**, **f**) (*P < 0.05; **P < 0.01; ***P < 0.001; ****P < 0.0001 between different plexuses within the same colonic region) (^#^*P* < 0.05; ^##^*P* < 0.01; ^####^*P* < 0.0001 between the same plexus in different colonic regions)

Quantitative data for the overall SP-IR neurons were cumulative and included data from preparations obtained from 17 animals, 7 preparations/animal. The highest density of SP-IR neurons/mm^2^ was observed in the ISP of both AC and DC (222.4 ± 10.2 vs. 166 ± 10.1), followed by the OSP (AC 51.1 ± 4.3 and DC 54.7 ± 4.6) and the MP (36.1 ± 2.7 and 47 ± 2.6 in AC and DC, respectively) (Fig. 1a). Moreover, there was a significant difference between ISP and OSP (*P* < 0.0001 in both AC and DC, and between ISP and MP (*P* < 0.0001 in both AC and DC as well as between ISP of AC and DC (*P* < 0.0001). The SP-IR neuronal population made up almost a third of HuCD-IR neurons in the ISP of both AC and DC, about 19–22% in the OSP and 13–17% in the MP with significant differences among the plexuses (*P* < 0.01–*P* < 0.0001) but not between the AC and DC (Fig. 1b).

**Fig. 2:**
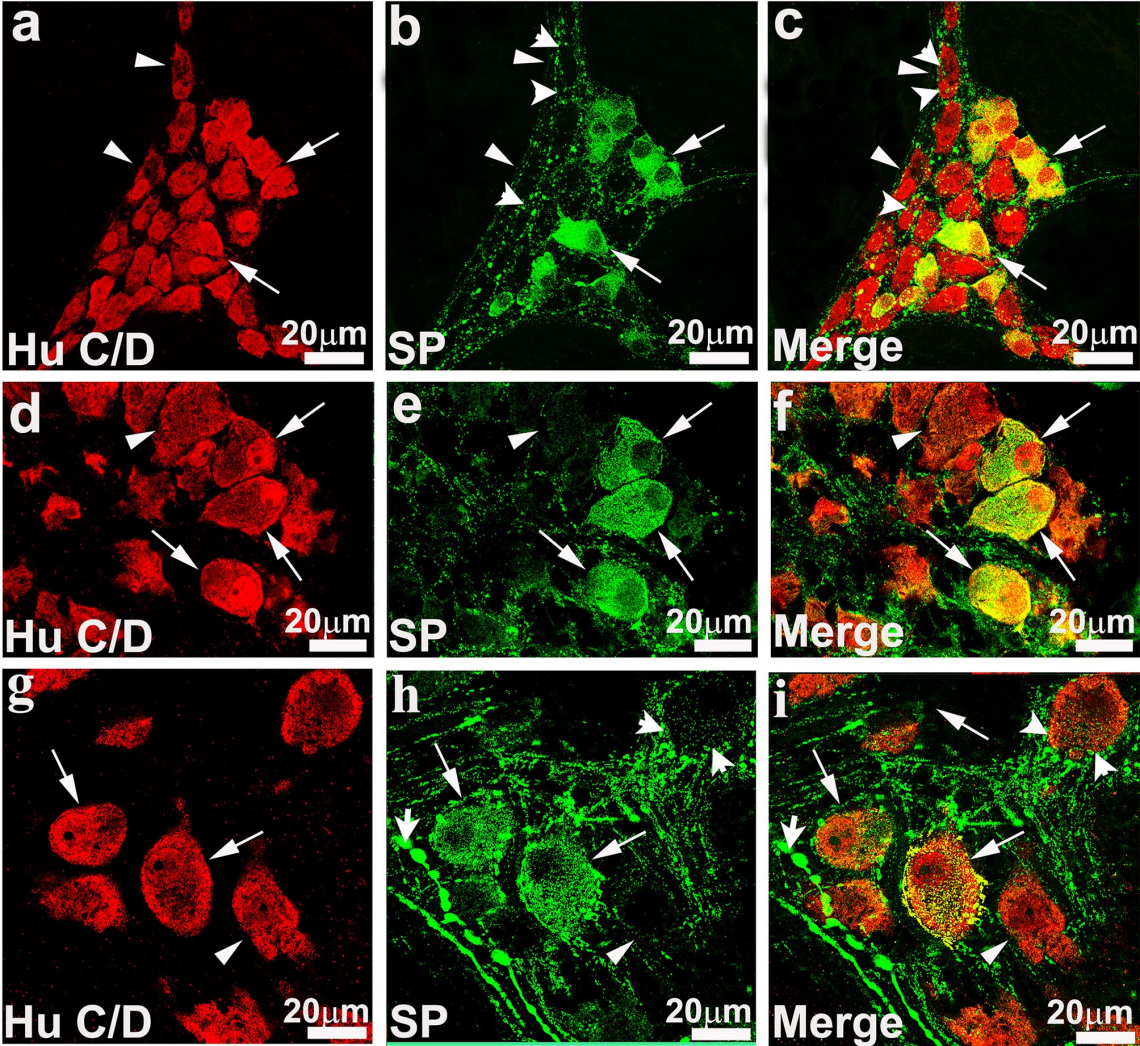
High-resolution confocal images of the submucosal (**a**–**c**, **d**–**f**) and myenteric (**g**–**i**) plexus of the porcine colon. **a** Immunofluorescence staining obtained with the pan neuronal marker HuC/D in the inner submucosal plexus of the descending colon. **b** Substance P (SP)-immunoreactive (-IR) neurons that exhibit an irregular, ovoidal soma profile (arrows) some of which with thick dendrites. SP-IR fibers in the ganglia show varicosities (arrowheads) and often form baskets around HuC/D neurons. **c** Overlap staining of HuC/D-IR and SP-IR. **d**–**f** Representative images of the outer submucosal plexus of the ascending colon. **d** HuC/D-IR neurons, **e** SP-IR neurons, and **f** merge staining. Arrows in **d**–**f** indicate examples of HuC/D/SP-IR neurons exhibiting rounded or oval profile. **g**–**i** Myenteric plexus of the ascending colon. Arrows point to neurons co-expressing HuC/D- (**g**) and SP-IR (**h**) (**i**, respective overlapping). SP-IR neurons displayed a variable size and morphology. SP-IR was detectable in thinner and thicker bundles of fibers (both exhibited varicosities) running around HuC/D-IR neurons (arrowheads)

The HuC/D antibody used to assess neuronal populations uniformly labeled the cytoplasm and often the nucleus of all neuronal cell bodies, but not neuronal processes (Fig. 2a, d, g). In contrast, SP immunostaining was observed in neuronal cell bodies (but not the nucleus) and neuronal processes that form bundles separating the ganglia (Fig. 2b, e, h). SP-IR fibers have numerous and prominent varicosities that often form baskets surrounding enteric neurons, which are particularly prominent in the MP (Fig. 2b, h). Different morphological types of neurons were observed in all plexuses, including neurons with elongated cell bodies and dendrites and neurons with ovoid and smooth-shaped cell bodies and long, thick axons (Fig. 2b, e, h).

### SP/ChAT expressing neurons

Quantitative data for SP/ChAT-IR neurons were obtained from the analysis of 46 whole mounts from 14 pigs. A substantial population of HuCD-IR neurons in the ISP coexpressed SP/ChAT-IR (164 ± 12/mm^2^ in AC and 121 ± 10/mm^2^ in DC, *P* < 0.01, representing ~ 21–23% of the entire neuronal population) (Fig. 1c, d). HuCD/SP/ChAT-IR neurons were less abundant in the OSP (AC 36.6 ± 5/mm^2^ and DC 41.5 ± 5.8/mm^2^, *P* < 0.0001 vs. ISP, corresponding to ~ 17% HuCD-IR neurons in both colonic regions) and even less in the MP (16.7 ± 2/mm^2^ and 31.3 ± 3.7/mm^2^, *P* < 0.0001 vs. ISP, corresponding to 7% and 12% of all neurons in AC and DC, respectively). The vast majority of HuCD/SP-IR neurons in each plexus of both AC and DC contained ChAT-IR ranging from 62 to 66% in the MP, 74 to 76% in the ISP, and 71 to 85% in the OSP (Suppl. Figure 1a). The SP-IR neurons made up about a third of the ChAT-IR neurons in the MP and OSP and about half of those in the ISP (Suppl. Figure 1b).

**Fig. 3:**
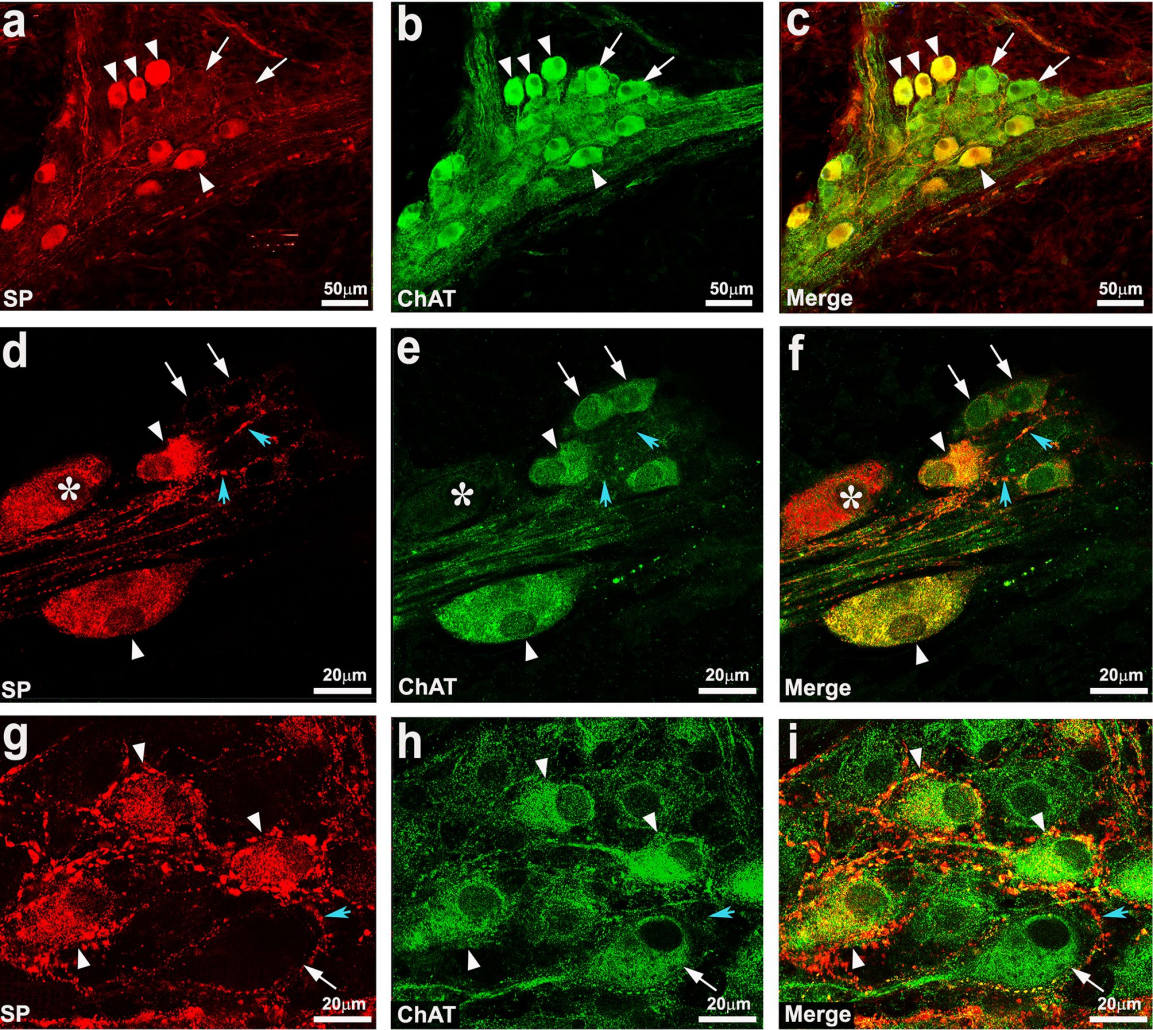
High-resolution confocal images. Double labeling of substance P (SP) and choline acetyltransferase (ChAT) immunoreactivities (IR) in ganglion cells in the outer submucosal plexus in the ascending colon (**a**–**c**) and in the descending colon myenteric plexus (**d**–**f**, **g**–**i**). **a**–**c** Arrowheads point to some ChAT-IR neurons containing SP-IR, whereas arrows point to examples of ChAT-IR neurons lacking SP-IR. **d**–**f** SP/ChAT-IR neurons (arrowheads), ChAT-IR neurons lacking SP-IR (arrows) as an example of a SP-IR neuron that does not contain ChAT-IR (asterisks). Blue arrowheads (**d**–**f** and **g**–**i**) indicate SP-IR (**f**): varicose fibers. **g**–**i** Some neurons co-expressing SP- and ChAT-IR (arrowheads) and a ChAT- IR neuron that does not contain SP-IR (arrows) and is surrounded by SP-IR fibers with varicosities. Blue arrowheads also point to prominent varicose fibers

Figure 3 shows examples of HuCD-IR containing SP- and ChAT-IR in the different plexuses in the AC and DC. Whereas most of the SP-IR also contained ChAT-IR, there are many ChAT-IR neurons lacking SP-IR, but there are also a few SP-IR neurons lacking ChAT-IR (Fig. 3 d–f). Both SP- and ChAT-IR are localized to fibers; ChAT-IR was predominantly localized to bundles of fibers, whereas SP-IR was predominantly localized to varicose fibers that often wrapped around enteric neurons suggesting the site of release (Fig. 3d, f, g, i).

### SP/nNOS-expressing neurons

For this data set, we analyzed 36 specimens from 13 pigs. There was a low density of neurons expressing HuCD/SP/nNOS-IR with the highest values in the ISP (AC 29.7 ± 3.1/mm^2^ and DC 50.7 ± 4.8/mm^2^, *P* < 0.0001) followed by OSP (AC 13 ± 3/mm^2^ and DC 18.9 ± 3/mm^2^, *P* < 0.01 vs. MP in AC and *P* < 0.0001 vs. ISP in DC) and MP (AC 6.4 ± 1.9 and DC 4.8 ± 1.2/mm^2^, *P* < 0.0001 vs. ISP in both AC and DC) (Fig. 1e). Overall, the SP/nNOS-IR neurons corresponded to < 10% of the neuornal populations visalized with HuCD-IR (Fig. 1f). SP/nNOS-IR neurons made up < 20% of the SP-IR neurons in the MP, 18–31% in the ISP, and 25–38% in the OSP (*P* < 0.01 vs. MP) (Suppl. Figure 2a). Similarly, SP/nNOS-IR neurons made up about a third of the overall nNOS neuronal population in the ISP, < 20% in the OSP (*P* < 0.05–0.01 vs*.* ISP) and < 10% in the MP (*P* < 0.0001 vs*.* ISP) (Suppl. Figure 2b).

**Fig. 4:**
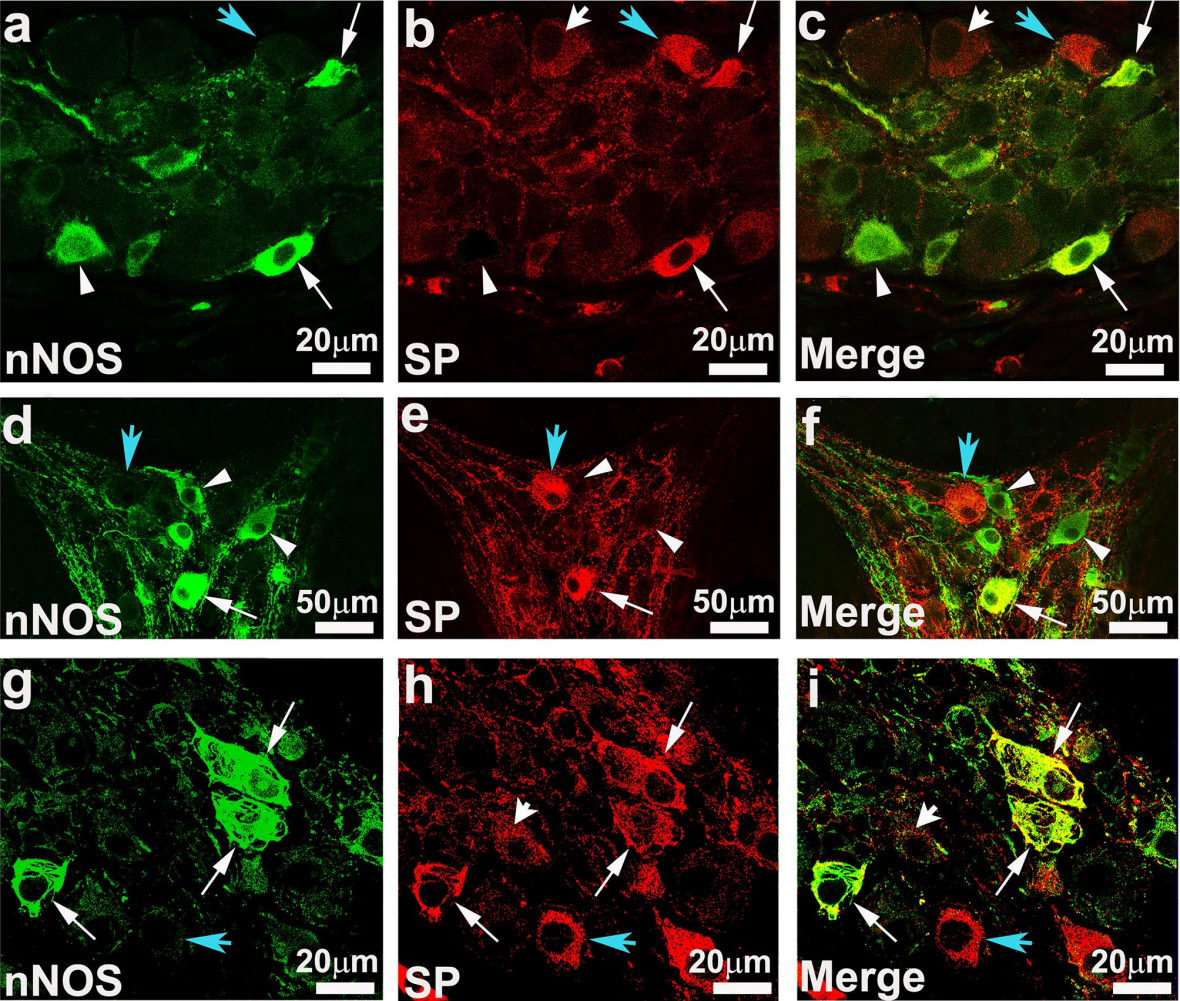
High-resolution confocal images showing staining for neuronal nitric oxide synthase (nNOS) (**a**, **d**, **g**) and SP (**b**, **e**, **h**) and merging of both staining (**c**, **f**, **i**) in the outer submucosal (**a**–**c** and **g**–**i**) and myenteric (**d**–**f**) plexus of the ascending colon. Arrows point to nNOS immunoreactive (-IR) neurons co-expressing SP-IR, while arrowheads indicate examples of nNOS-IR neurons lacking SP-IR and blue arrowheads point to neurons that do not contain SP-IR. Some nNOS-IR neurons showed cellular projections (dendrites or axons) emerging from the cell body and size and morphology variability

Figure 4 shows examples of HuCD/SP-IR containing nNOS-IR in the different plexuses in the AC and DC, as well as HuCD-IR neurons containing nNOS but lacking SP and HuCD-IR neurons positive for SP but not nNOS. Some nNOS-IR neurons showed cellular projections (dendrites or axons) emerging from the cell body and variability in size and morphology of their cell body. SP- and nNOS-IR are also observed in nerve fibers, with the SP-IR fibers being varicose and surrounding cell bodies.

### SP/ChAT/nNOS-expressing neurons

**Fig. 5:**
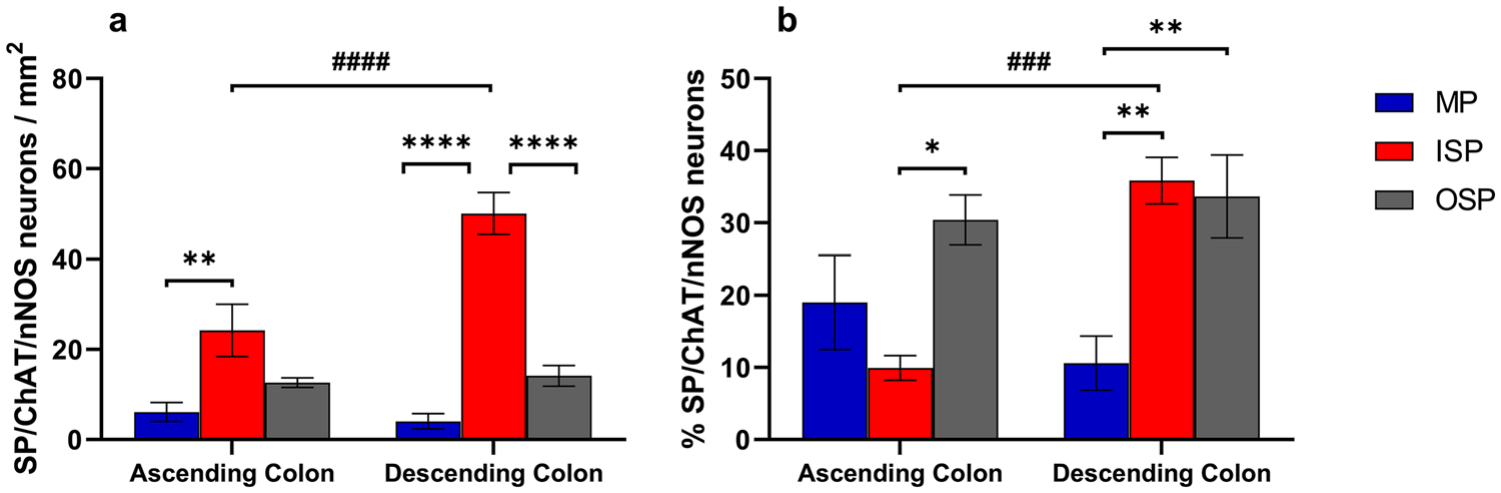
Graphs showing the density of neurons containing SP/ChAT/nNOS-IR (**a**) in the inner submucosal plexus (ISP), outer submucosal plexus (OSP), and myenteric plexus (MP) of the ascending and descending colon expressed as numbers of neurons/mm^2^. The graph in **b** shows the % of SP-IR neurons that contain both ChAT- and nNOS-IR (*P < 0.05; **P < 0.01; ****P < 0.0001 between different plexuses within the same colonic region) (^###^*P* < 0.001; ^####^*P* < 0.0001 between the same plexus in different colonic regions)

Quantitative data for the SP/ChAT/nNOS-IR neurons were obtained from 30 specimens from 13 animals. Enteric neurons containing all three transmitters/modulators were observed with the highest density in the ISP (24.2 ± 5.8/mm^2^ in the AC vs. 50 ± 4.6/mm^2^ in the DC, *P* < 0.0001) followed by the OSP (12.6 ± 1/mm^2^ in AC and 14.2 ± 2.3/mm^2^ in DC, *P* < 0.0001 vs*.* ISP) and the lowest density in the MP (6.1 ± 2/mm^2^ in AC, *P* < 0.01 vs*.* ISP and 4 ± 1.7/mm^2^ in DC, *P* < 0.0001 vs. ISP) (Fig. 5a). SP/ChAT/nNOS-IR neurons represented < 20% of the SP-IR neurons in the MP and about a third of SP-IR neurons in the OSP (Fig. 5b), whereas in the ISP, they made up 10% of SP-IR neurons in the AC and 36% in the DC, *P* < 0.001) (Fig. 5b).

**Fig. 6:**
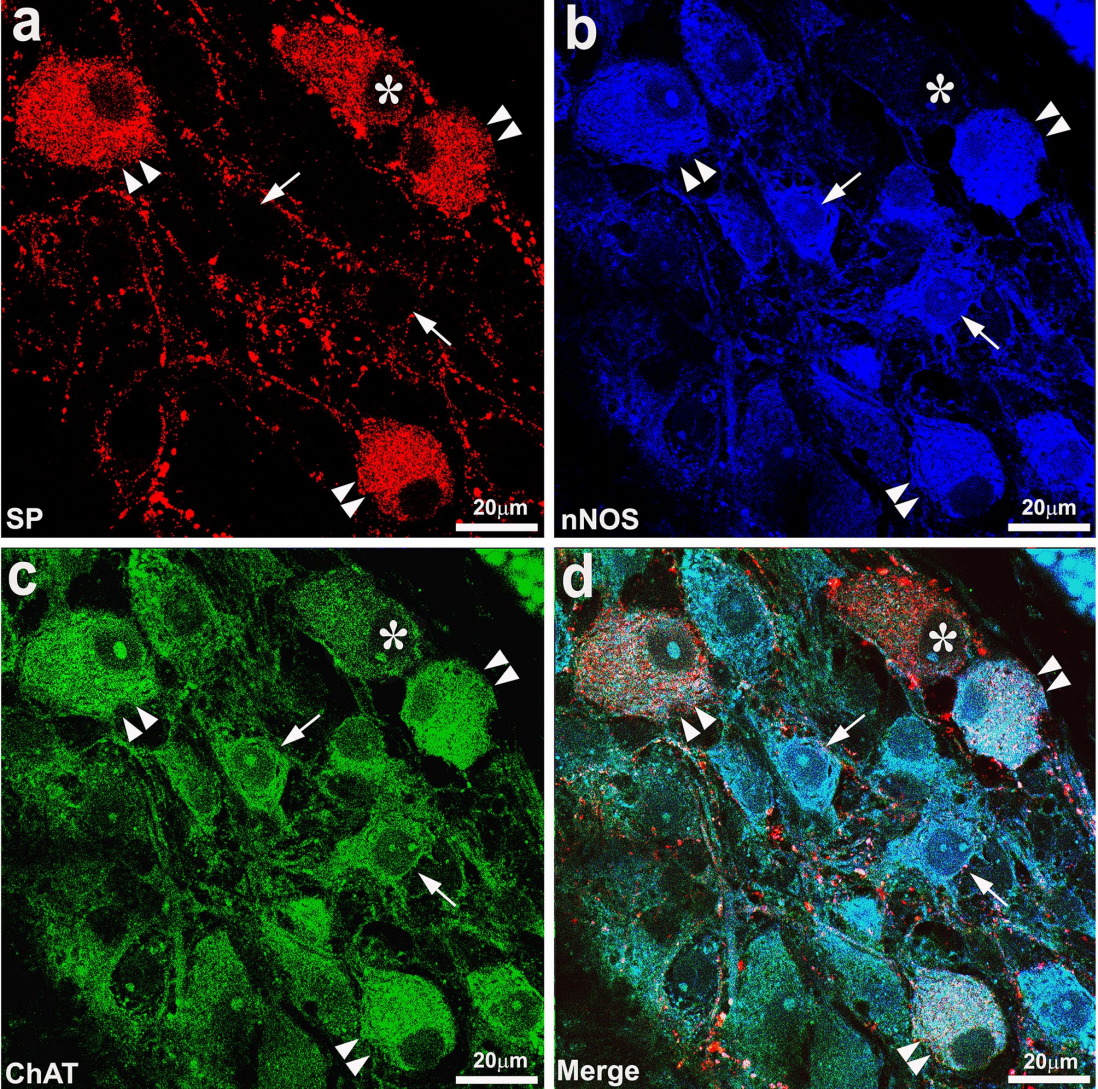
High-resolution confocal images showing colocalization of SP- (**a**), nNOS- (**b**), and ChAT-immunoreactivity (IR) (**c**). **d** The overlay of SP/nNOS/ChAT-IRs. Double arrowheads point to SP/nNOS/ChAT-IR neurons, arrows point to nNOS/ChAT-IR lacking SP-IR, whereas asterisks indicate a SP-IR neuron positive for ChAT-IR but negative for nNOS-IR

Figure 6 shows examples of colocalization of SP-, ChAT-, and nNOS-IR in enteric neurons of ISP and MP of the AC and DC. SP/ChAT/nNOS-IR neurons were also observed in the OSP of both AC and DC (not shown).

## Discussion

This study shows that SP-IR neurons represent about a third of the enteric neurons visualized by the pan-neuronal marker HuCD (Lin et al. 2002; Phillips et al. 2004) in the ISP of the porcine colon with similar density in the AC and DC, whereas they are significantly less abundant in the OSP and MP in both AC and DC. The vast majority of the SP-IR neurons contains the excitatory marker, ChAT, with little differences between plexuses and colonic regions, whereas the proportion of SP-IR neurons co-expressing the inhibitory transmitter nNOS is much lower. The proportion of SP-IR neurons containing both ChAT- and nNOS-IR varies in different plexuses with the highest density in the ISP of DC followed by the OSP in both AC and DC, and the lowest density in the ISP of AC followed by the MP of both AC and DC. This is the first systematic study focused on the distribution, quantification, and neurochemical characterization of SP-IR enteric neurons in the different plexuses of the porcine AC and DC.

SP-IR has been identified in enteric neuronal perikarya within intramural plexuses and in nerve processes in the GI tract of different mammalian species, including the pig (Timmermans et al. 1989; Schmidt et al. 1991; Hens et al. 2000; Brookes 2001; Brehmer et al. 2004; Mitsui 2011; Gonkowski 2013; Petto et al. 2015; Filmayer et al., 2020). SP exerts a wide array of regulatory effects according to the species, segment of the GI tract and type of neurokinin (NK) receptors that are activated (Holzer and Holzer-Petsche 2001; Tonini et al. 2001; Lecci et al. 2006). One of SP’s main effects is to evoke contraction of intestinal smooth muscles (Holzer and Holzer-Petsche 1997a). SP also regulates gastric and intestinal secretion (Lecci et al. 2006; Holzer and Holzer-Petsche 1997a,b; Shimizu et al. 2008) and blood flow (De Fontgalland et al. 2008) and plays a key role in inflammatory processes through its action on lymphocytes and macrophages (Mantyh et al. 1994) and by promoting the release of various proinflammatory factors (Holzer and Holzer-Petsche 1997a,b; Zhao et al. 2002; Shimizu et al. 2008).

In pigs, SP distribution has been reported in the small intestine, the piglet colon, and the adult distal colon (Timmermans et al., 1989; Schmidt et al. 1991; Petto et al 2015; Gonkowski 2013; Filzmayer et al. 2020). Our study differs from previous investigations in that it focused on the quantification and neurochemical characterization of SP-enteric neurons in different regions of the adult porcine colon. The highest density of SP-IR neurons (the number of neurons/mm^2^ and the % of HuCD/SP neurons) detected in the ISP is in agreement with that reported by Gonkowski (2013) in the porcine DC. In addition, in line with our results, Petto et al. (2015) found a larger number of SP-positive neurons in the ISP compared to the OSP in wholemounts of piglet proximal colon. Furthermore, using neuronal tracing, Hens et al. (2000) visualized the highest number of DiI labeled/SP-positive neurons in the ISP, followed by the OSP and MP of the pig small intestine. By contrast, our findings of a comparable percentage of SP-IR neurons in the ISP (27–28%) and OSP (19–22%) in the AC and DC appear to be at variance with the data in Petto et al. (2015) reporting similar percentages of SP-positive neurons in the ISP (32.7%) but lower percentage in the OSP (11.9%). Although our study and that of Petto et al. (2015) are based on similar immunohistochemical approaches (i.e., double labeling with SP and HuCD), the different percentages can be ascribed to different factors, including animal age (adult pigs vs. unweaned piglets) and the number of examined ganglia (individual vs*.* randomly selected ISP, OSP, and MP colonic ganglia). Overall, from our and previously reported data, the density of SP-IR neurons appears to be ISP > OSP >  > MP.

The considerable number of SP-IR submucosal neurons, especially in the ISP that lies near the mucosa, supports sensory and secretomotor functions of SP in the porcine colon (Timmermans et al. 1997; Hens et al. 2000). Specifically, SP-IR neurons in the ISP likely function as secretomotor neurons in both small and large mammals (Bornstein et al. 1989; Brown et al. 1992; Timmermans et al. 1997). Submucosal SP-IR neurons have also been shown to contribute to the motor innervation supplying the pig small intestine muscle (Hens et al. 2002).

We have previously shown that ChAT-IR neurons represent a large proportion of HuCD neurons ranging from 45 to 55% n the MP, 40 to 45% in the OSP, whereas they were less abundant (< 35%) in the ISP (Mazzoni et al. 2021). In the present study, we found that SP and ChAT-IR make up > 20% of the entire HuCD neuronal population in the ISP and 17% in the OSP with the lowest density in the MP (≤ 12%). Whereas the vast majority of SP-IR neurons (up to 85%) are cholinergic, we observed SP-IR neurons that do not contain ChAT-IR (see Fig. 2), unlike the study of Petto et al. (2015) which reported no SP-IR neurons without ChAT. On the other hands, SP-IR neurons make up a major population of ChAT-IR neurons (from a third to half, depending on the plexus and the region). ChAT/SP-IR neurons represent the classic combination of excitatory neurotransmitters expressed in motor neurons that innervate the circular and longitudinal muscular layer (Brookes et al. 1991; Steele et al. 1991). In the guinea pig, a proportion of myenteric ChAT/SP-positive neurons project to the mucosa (Song et al. 1992; Neunlist and Schemann 1997) and are likely to function as intrinsic sensory neurons responding to stretch and various chemical stimuli (Furness et al. 1998; Li and Furness 1998; Neunlist et al. 1999). In the human colon, DiI tracing studies in the MP revealed that ~ 60% of neurons with ascending projections were positive for enkephalin (ENK) and about one-third of the ENK-IR neurons were also SP-IR (Humenick et al., 2021). In this contest, Humenick et al. (2021) suggested that in the human colon, there is a class of ascending interneurons expressing ChAT/ENK/SP similarly to the guinea pig distal colon where multiple ascending interneurons have been distinguished (Lomax and Furness 2000).

In the ISP of the proximal and distal porcine colon, Filzmayer et al. (2020) reported that mechanosensitive enteric neurons and stretch-sensitive enteric neurons were predominanly positive for ChAT and more than half where ChAT/SP. Notably, numerous SP-IR nerve processes originating from the ISP ganglia travel towards the mucosa and down to deeper layers of the gut wall. Similar to acetylcholine, SP is a powerful secretagogue in the pig GI tract (Blumenthal et al. 1998; Frieling et al. 2000; Pfannkuche et al. 2011). The high percentage of SP/ChAT-IR neurons in the ISP suggests various neuronal phenotypes, including cholinergic secretomotor neurons (projecting to the mucosa) (Brown and Timmermans 2004) and interneurons (Petto et al. 2015), as indicated by the dense SP-IR neuropil within the ganglia, which is particularly abundant in the MP. Physiologically, SP/ChAT may regulate secretion and related absorptive functions both directly, by exerting a prosecretory action on colonocytes, and indirectly, by inhibiting other submucosal neurons (Riegler et al. 1999; 2000; Pfannkuche et al. 2011).

In this study, we also found colocalization of SP and the inhibitory transmitter nNOS. SP/nNOS neurons represent a small proportion of the entire HuCD neuronal population, with the highest density in the ISP of the DC (< 10% of HuCD-IR neurons) and the lowest density in the MP (< 3% of HuCD-IR neurons). SP/nNOS-IR neurons represent up to 35% of the SP-IR neuronal population in the AC OSP with the lowest percentage in the MP (18–20%) and the ISP (16%). nNOS/SP-IR enteric neurons have been identified in various segments of the mammalian GI tract, including the pig (Bulc et al. 2019), sheep pylorus and ileum (Mazzuoli et al. 2007; 2008), and humans’ antrum and colon (Pimont et al. 2003; Yuan et al. 2021), whereas an earlier study reported no SP-IR in nitrergic neurons visualized with the reduced nicotinamide adenine dinucleotide phosphate (NADPH)-diaphorase activity method (Keränen et al 1995). The different degrees or lack of colocalization of these transmitters/modulators previously reported likely depend on the GI segment and species and methodology used. nNOS-IR neurons comprise inhibitory motor neurons to both the longitudinal and circular muscle layers and descending inhibitory interneurons in different species, including the pig (Sanders and Ward 2019; Murphy et al. 2007; Timmermans et al. 2001; Brown and Timmermans 2004), thus is reasonable to speculate that SP/nNOS-IR neurons in the pig colon include these neuronal phenotypes, as suggested by Mazzuoli et al. (2008) for nNOS- and SP-IR neurons in the MP of the sheep pylorus. In addition, the localization of SP/nNOS-IR neurons in the ISP, together with the finding of nNOS-IR fibers in the mucosa (Pfannkuche et al. 2011), support that they include secretomotor neurons.

As expected on the basis of the quantitative analysis performed in this study, a small population of neurons contained all three neurotrasnmitters/modulators, which was more abundant in the OSP of DC and quite sparse in the other plexuses and region with the lowest density in the MP. Though the functional significance of these neuronal subsets remains to be established, on the basis of the functional roles of each of these transmitters and the neuronal localization, it is likely that they include motor neurons, secretomotor neurons, interneurons, and perhaps also primary afferent neurons (Brown and Timmermans 2004).

In conclusion, this study provides a comprehensive analysis of the density and neurochemical characterization of SP-IR neurons in each plexus of two distinct regions of the adult pig colon. We identified neurochemically distinct populations of neurons: SP/ChAT, SP/nNOS, SP/ChAT/nNOS, as well as neurons that only contain ChAT or nNOS or SP, though the latter are a small component. These different neuronal subsets reflect functionally distinct populations, likely including excitatory and inhibitory motorneurons to the lingitudinal and circular muscle, ascending excitatory and descending interneurons, secretomotor neurons to the mucosa, and possibly primary afferent neurons. In this study, we have not performed an in-depth analysis of the shape, size, and morphology of the SP-IR neurons and of the distribution of their terminals; thus, we cannot state whether there were differences in this respect among the distinct SP populations. The different density and neurochemical profile might reflect different functions of SP-IR neurons such as immune modulation, regulation of mucosal function, and control of vascular, myogenic, and neurogenic activities, though additional studies are required to establish this concept. The pig has been gaining increasing interest as a valuable animal model for studying intestinal functions and disorders, for its structural and functional homologies with humans, including the size that makes it amenable to study surgical procedures and drug development, as well as for for genomic and chromosomal similarities and microbial species and metabolic processes (Litten-Brown et al. 2010; Gonzales et al. 2015). These many homologies make the pig an important translational model to study human health and disease. Mapping neuromodulators’ expression in a pre-clinical model provides the basis for elucidating neuronal circuits underlying GI functions and advancing our understanding of health and diseases in humans.

### Supplementary Information

Below is the link to the electronic supplementary material.

Supplementary file1 (TIF 24650 KB)

Supplementary file2 (TIF 24578 KB)

Supplementary file3 (DOCX 17 KB)

## Data Availability

The authors state that supporting data of the findings of this study are available within the main text of the paper, and in the supplementary materials section.
