# Supplementary figures and images for: Distribution, quantification, and characterization of substance P enteric neurons in the submucosal and myenteric plexuses of the porcine colon
Source: Cell Tissue Res. 2023 Nov 20;395(1):39–51. doi: 10.1007/s00441-023-03842-x (PMC10774220; doi:10.1007/s00441-023-03842-x)

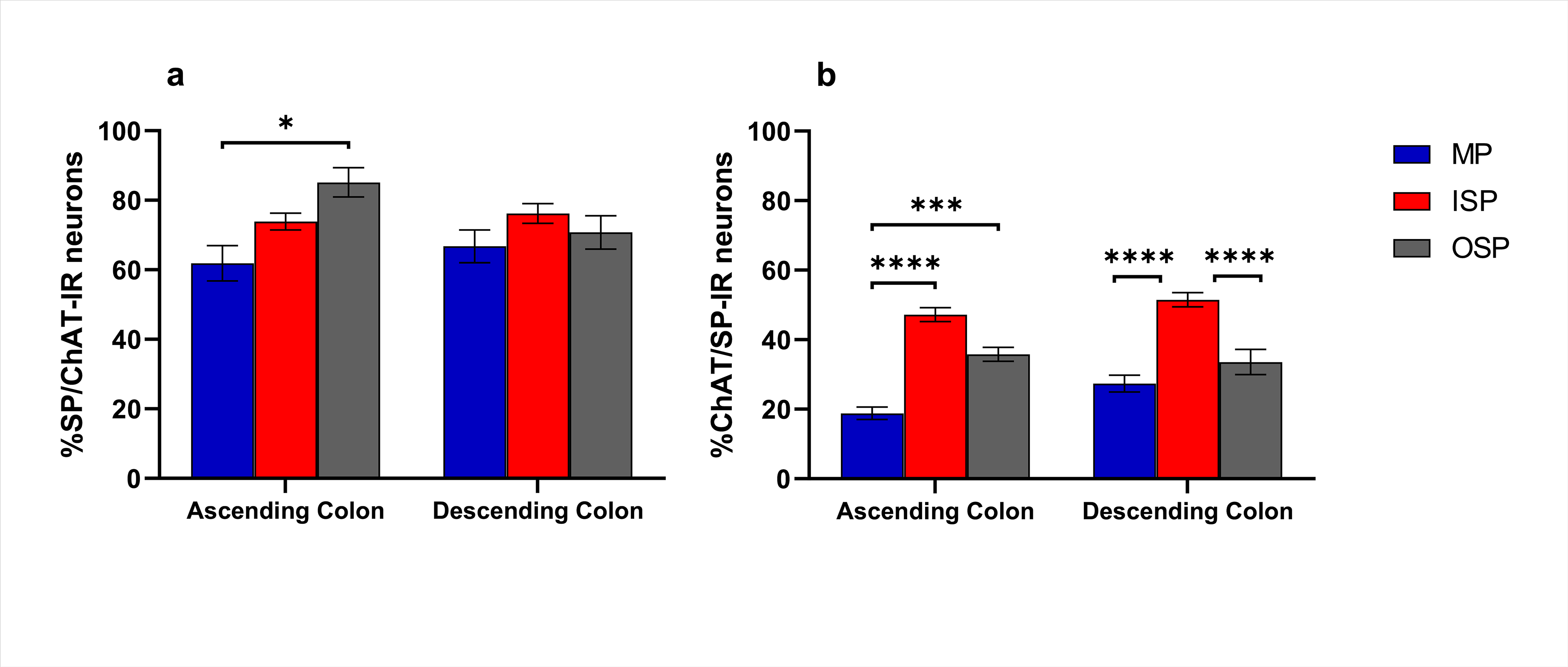

Supplement: Supplementary file 1 — Supplementary file1 (TIF 24650 KB) [file 441_2023_3842_MOESM1_ESM.tif]

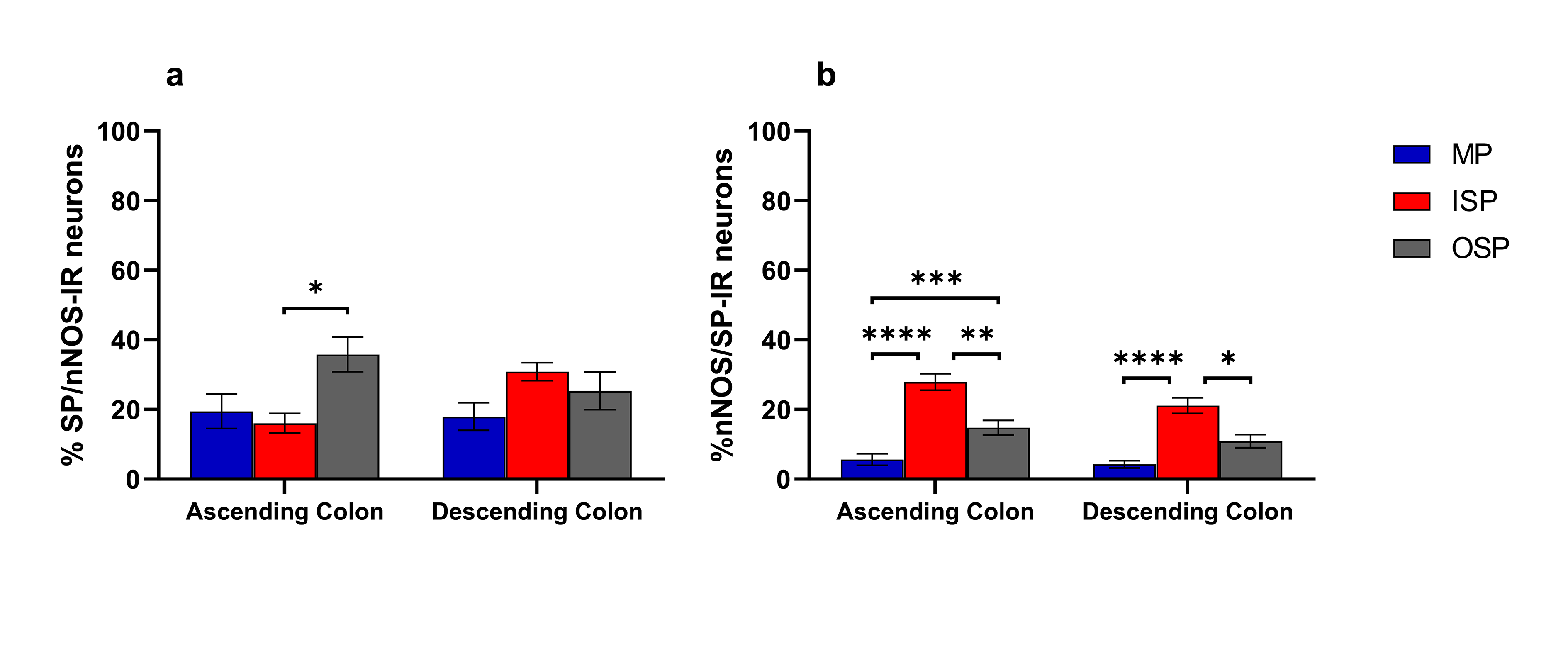

Supplement: Supplementary file 2 — Supplementary file2 (TIF 24578 KB) [file 441_2023_3842_MOESM2_ESM.tif]
