# Supplementary material for: Distribution, quantification, and characterization of substance P enteric neurons in the submucosal and myenteric plexuses of the porcine colon
Source: Cell Tissue Res. 2023 Nov 20;395(1):39–51. doi: 10.1007/s00441-023-03842-x (PMC10774220; doi:10.1007/s00441-023-03842-x)
Supplement: Supplementary file 3 — Supplementary file3 (DOCX 17 KB) [file 441_2023_3842_MOESM3_ESM.docx]

**Distribution, quantification and characterization of substance P enteric neurons in the submucosal and myenteric plexuses of the porcine colon**

Maurizio Mazzoni^1+^, Luis Cabanillas^2,3+^, Anna Costanzini^4^, Filippo Caremoli,^2&^

Mulugeta Million^2,5^, Muriel Larauche^2^, Paolo Clavenzani,^1^

Roberto De Giorgio^4^ and Catia Sternini^2,3,*^

**Supplemental figures legends**

**Supplemental Fig. 1.** Graphs showing the % of SP-IR neurons containing ChAT-IR (**a**) and the % of ChAT-IR neurons containing SP-IR (**b**) in the inner submucosal plexus (ISP), outer submucosal plexus (OSP) and myenteric plexus (MP) of the ascending and descending colon. * P<0.05; *** P<0.001; **** P<0.0001 between different plexuses within the same colonic region; there were no statistically significant differences between the same plexus in different colonic regions.

**Supplemental Fig. 2.** Graphs showing the % of SP-IR neurons containing nNOS-IR (**a**) and the % of nNOS-IR neurons containing SP-IR (**b**) in the inner submucosal plexus (ISP), outer submucosal plexus (OSP) and myenteric plexus (MP) of the ascending and descending colon. * P<0.05; ** P<0.01; *** P<0.001; **** P<0.0001 between different plexuses within the same colonic region; there were no statistically significant differences between the same plexus in different colonic regions.
